# Mutations in *bdcA* and *valS* Correlate with Quinolone Resistance in Wastewater *Escherichia coli*

**DOI:** 10.3390/ijms22116063

**Published:** 2021-06-04

**Authors:** Negin Malekian, Ali Al-Fatlawi, Thomas U. Berendonk, Michael Schroeder

**Affiliations:** 1 Biotechnology Center (BIOTEC), Dresden University of Technology, Tatzberg 47-49, 01307 Dresden, Germany; negin.malekian@tu-dresden.de (N.M.); ali.al-fatlawi@tu-dresden.de (A.A.-F.); 2 Institute of Hydrobiology, Dresden University of Technology, 01217 Dresden, Germany; thomas.berendonk@tu-dresden.de

**Keywords:** *E. coli*, quinolones, antibiotic resistance, genome-wide association study (GWAS)

## Abstract

Single mutations can confer resistance to antibiotics. Identifying such mutations can help to develop and improve drugs. Here, we systematically screen for candidate quinolone resistance-conferring mutations. We sequenced highly diverse wastewater *E. coli* and performed a genome-wide association study (GWAS) to determine associations between over 200,000 mutations and quinolone resistance phenotypes. We uncovered 13 statistically significant mutations including 1 located at the active site of the biofilm dispersal gene *bdcA* and 6 silent mutations in the aminoacyl-tRNA synthetase *valS*. The study also recovered the known mutations in the topoisomerases gyrase (*gyrA*) and topoisomerase IV (*parC*). In summary, we demonstrate that GWAS effectively and comprehensively identifies resistance mutations without a priori knowledge of targets and mode of action. The results suggest that mutations in the *bdcA* and *valS* genes, which are involved in biofilm dispersal and translation, may lead to novel resistance mechanisms.

## 1. Introduction

In the 1960s, an impurity during the synthesis of the antimalarial chloroquine led to the discovery of nalidixic acid [1,2]. Two years after its introduction to the market, resistances were observed, but it took even more years before the drug’s targets and mechanism of action were understood [1,3]. In 1964 and 1990, gyrase (*gyrA*) and topoisomerase IV (*parC*) were discovered as the drug’s primary and secondary targets, respectively [1]. Subsequently, improved derivatives of nalidixic acid were found, such as norfloxacin, ciprofloxacin, and then levofloxacin. Today, there are over 20 fluoroquinolones on the market. Generally, fluoroquinolones act by converting their targets, gyrase (*gyrA*) and topoisomerase IV (*parC*), into toxic enzymes that fragment the bacterial chromosome [4]. With the wide use of quinolones, however, bacteria developed resistances through several routes such as increased expression of efflux pumps, which transport drugs outside the bacterial cell, or horizontal gene transfer of resistance genes, whose gene products bind to the quinolone targets [4]. However, the most direct route to resistance is mutations in the drug targets *gyrA* and *parC*. Specifically, changes in the amino acids Ser83 and Asp87 of *gyrA* and Ser80 of *parC* confer resistance [4,5] to quinolones.

The discovery of these mutations was driven by a deep understanding of the mechanism of action of quinolones. Already over 50 years ago, Crumplin et al. suggested that “a comparative study of […] mutants and otherwise isogenic bacteria should facilitate identification of the hitherto unknown […] target” [3], which was at the time not possible on a genome-wide scale. This changed with the advent of deep sequencing technology. Thus, we want to complement the original hypothesis-driven approach to understand resistance [3] with a hypothesis-free, high-throughput approach, in which we systematically evaluate the mutational landscape of resistant and susceptible bacteria. In the other words, we screen entire bacterial genomes of many isolates and correlate them to patterns of the isolates’ susceptibility and resistance. This approach termed genome-wide association study, GWAS, rose with the advent of deep sequencing and was initially applied to human genomes and disease phenotypes [6]. Recently, the success of human GWAS sparked interest in microbial GWAS [7,8]. Genome-wide associations in bacteria are challenging, as clonal reproduction in bacteria leads to a nonrandom association of alleles at different loci (linkage disequilibrium (LD)) and population structure [8,9].

As an example for the dependencies of loci (linkage disequilibrium), the mutations in *gyrA* and *parC* correlate with each other, as they belong to the same resistance mechanism. However, following terminology from cancer biology, all of them are driver mutations, which cause clonal expansion in contrast to passenger mutations, which do not influence the fitness of a clone [10]. Driver mutations may impact clonal expansion directly by changing the amino acid sequence (nonsynonymous mutations) and thus protein structure or function, or they may act indirectly as synonymous mutations without changes to the amino acid sequence. Synonymous mutations may affect splicing, RNA stability, RNA folding, translation, or cotranslational protein folding [11]. Kimchi et al. showed that a synonymous mutation in the multidrug resistance gene MDR1 altered drug and inhibitor interactions [12]. Thus, a genome-wide association study aiming to uncover novel resistance mechanisms should consider both nonsynonymous and synonymous mutations, whose loci are not in linkage disequilibrium with those of already known mechanisms.

The population structure of *E. coli* is predominantly clonal, allowing the delineation of major phylogenetic groups, the largest being A (40%), B2 (25%), and B1 and D (both 17%) [13]. Therefore, any model of a genome-wide association study in *E. coli* should accommodate these groups. Interestingly, the groups also relate to pathogenicity: commensal *E. coli*, as e.g., found in human intestines, are more likely to belong to A and B1, and pathogenic *E. coli* are more likely to belong to B2 and D.

Generally, *E. coli* genomes vary in size between 4000 and 5500 genes, of which only half are shared by all *E. coli* [14]. These genes, which are common to all *E. coli*, define the core genome. In contrast to the core genome, the pan-genome is defined as the entire set of genes in a population appearing at least in one genome. The *E. coli* pan-genome exceeds 13,000 genes and has possibly no limit due to the bacteria’s ability to absorb genetic material [14]. Besides the pan-genome and core genome, Chattopadhyay et al. [15] used the term “core variome” to refer to the core genes’ variome, for *E. coli* and *Salmonella*. Additionally, in a nonbacterial context, the term “pan-cancer variome” is used to refer to the variomes shared by several types of cancer [16]. However, we define the pan-variome and core-variome in a manner similar to the pan-genome and core-genome. The former is defined as the mutations shared by all genomes, and the latter refers to the mutations present in at least one genome. Mutations correlating with resistance will—by definition—not be part of the core-variome. Hence, it is important for a genome-wide association study that there is a significant gap in size between core-variome and pan-variome.

*E. coli* pathotypes are well recognized as one of the major sources of human infection. Their effectiveness as pathogens has been linked to their development of antibiotic resistance. To date, it is not fully understood, how antibiotic resistance develops. It is ancient and inherent to bacteria [17] and can therefore be found in the natural environment. However, with the wide use of antibiotics, major sources of resistant bacteria are clinics and wastewater [18]. In particular, the latter plays an important role, since treatment plants act as melting pots for bacteria of human, clinical, animal, and environmental origin [18]. The high genetic diversity of a clinical *E. coli* population was substantially exceeded by a wastewater population [19], which makes wastewater *E. coli* a suitable source for a GWAS analysis.

**Figure 1:**
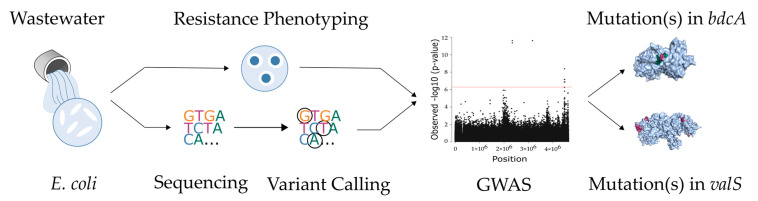
Wastewater *E. coli* were phenotyped and sequenced. Variants were called and correlated to quinolone resistance in a GWAS study resulting in novel candidate resistance mutation.

In this study, we collected 1178 *E. coli* isolates from the municipal wastewater treatment plant in Dresden, Germany. The resistance of these isolates against 20 antibiotics, including quinolones, was measured using the agar diffusion method. Finally, 103 sequences that are representative in terms of resistance for the 20 antibiotics were sequenced. In our previous work, Mahfouz et al. [19] correlated genes in this dataset with a resistance phenotype. However, here, we looked for the variants associated with the resistance. To do so, we employed a computational approach and implemented variant calling on these genomes and then determined associations between the identified mutations and resistance levels of four quinolones covering first to third generations (nalidixic acid, norfloxacin, ciprofloxacin, and levofloxacin). We also considered population structure and dependencies among mutations. Building on the *gyrA* and *parC* mutations as positive controls, we characterized the quantity and quality of the mutational resistance landscape. We investigated whether there are resistance mutations beyond the ones in *gyrA* and *parC* and whether they may open new avenues for future drug discovery. In summary, we aimed to show that a bacterial genome-wide association study can effectively and comprehensively identify targets relevant to antibiotic resistance (see Figure 1).

## 2. Results

We aimed to identify mutations that correlate with quinolone resistance. After extracting raw variants from 99 wastewater *E. coli* genomes, we reduced raw to high-quality variants. We also evaluated the variome diversity of our samples (the pan-variome analysis) as a prerequisite for GWAS. Next, we explored the population structure of our samples to be considered in our GWAS. Then, we applied association analysis between variants and antibiotic resistance phenotypes to reduce high-quality to highly significant variants. The highly significant variants consist of the known mutations in *gyrA* and *parC* (our positive control) and some novel synonymous and nonsynonymous mutations. Next, we verified that the loci of new mutations are not in linkage disequilibrium with those of positive control. Finally, we looked into the biological function of the genes with novel variants. For the novel nonsynonymous mutation, we investigated its 3D structure and also checked its frequency among other antibiotics as well as the complete *E. coli* genomes available from the NCBI and other Gammaproteobacteria from Eggnog.

### 2.1. From Raw to High-Quality Variants

From the genomes, we extracted 457,554 raw variants, which we subjected to quality control steps resulting in 206,633 high-quality variants. Filtering rare variants, which appear in less than 5% of isolates, led to the greatest reduction in mutations of nearly 50% (see Table 1). This is an indication of a big gap between the pan-variome and core variome, which we discuss next.

### 2.2. Pan-Variome and Core Variome

**Figure 2:**
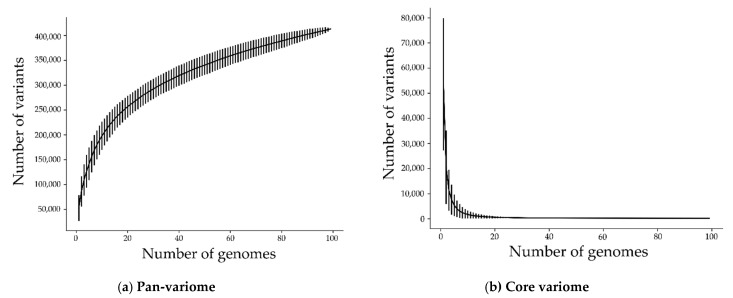
(**a**) Pan-variome (union of variants) and (**b**) core-variome (intersection of variants) of 206,633 high-quality and 206,650 rare variants (413283 in total). The standard deviation is added as error bars around the mean value for 1000 iterations. Most variants appear only in a few of the isolates.

For a genome-wide association study, it is vital that the mutations spread across the isolates. To characterize the distribution and diversity of the high-quality mutations, we computed the core-variome and the pan-variome (see Figure 2). The core-variome reflects the number of variants shared by a given number of genomes. In contrast, the pan-variome is the number of variants that exist in at least one genome within the given number of genomes, thus reflecting the total diversity of variants present in all genomes. As expected, the pan-variome grows fast, and the core-variome tails off fast. As seen in the same figure, for 20 genomes, the pan-variome consists already of some 256,000 variants, while the core-variome is reduced to some 600 variants. This means that there are only very few variants that are shared across many or even all of the genomes. Similarly, the graph for the pan-variome continually grows. Each added genome contributes new variants until the pan-variome reaches 413,283 variants (206,633 high-quality plus 206,650 rare variants) in total. Overall, the distribution of variants is thus suitable for GWAS as the core-variome and pan-variome are significantly different in size.

### 2.3. Phylogenetic Groups and Population Structure

**Figure 3:**
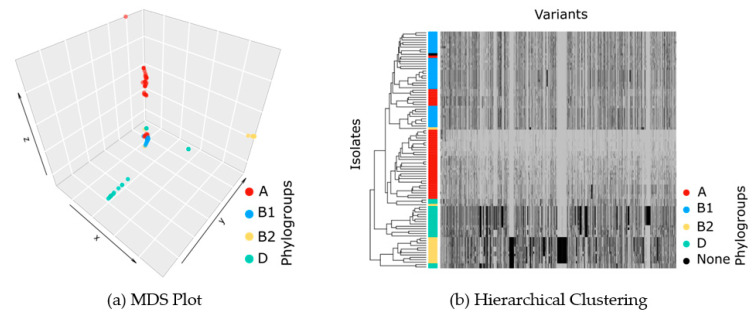
(**a**) Multidimensional scaling plot (MDS) on distances between isolates, calculated based on high-dimensional vectors of all mutations. Four clusters are found, which reflect the population structure in the GWAS model and which broadly coincide with phylogroups A, B1, B2, and D. (**b**) Hierarchical clustering on the vectors of presence/absence of variants for different isolates, where the presence of a variant is shown by black and its absence by gray.

A key ingredient of the GWAS model is the population structure. We applied MDS on distances between isolates, calculated based on high-dimensional vectors of all mutations, as well as hierarchal clustering on the vectors of presence and absence of variants. We identified four clusters (Figure 3), which broadly correspond to phylogenetic groups A, B1, B2, and D. Thus, our GWAS model correctly caters to the main *E. coli* lineages. After applying GWAS, we assessed the control of our study over the population structure using QQ plots.

### 2.4. From High-Quality to Highly Significant Variants

**Figure 4:**
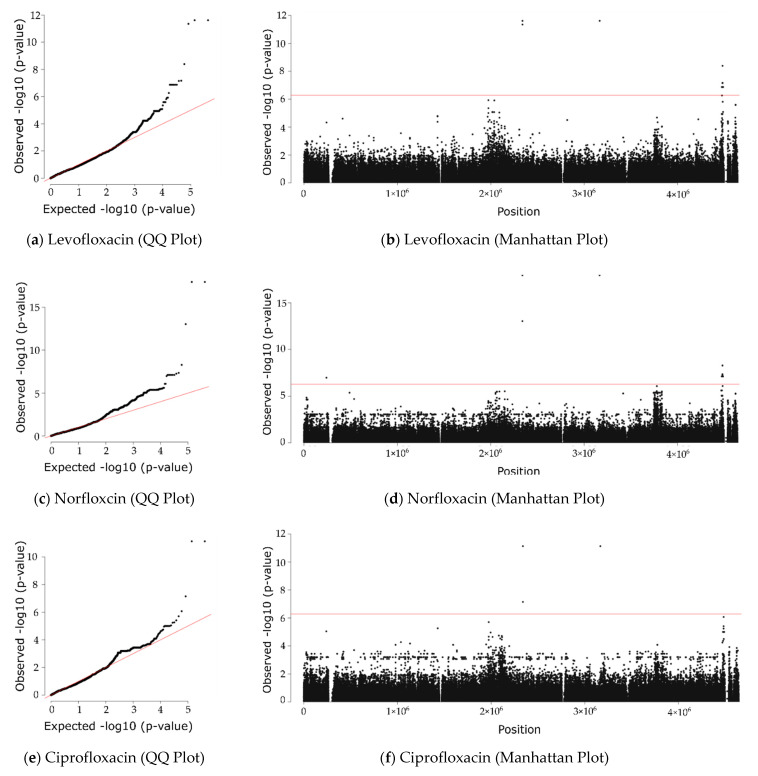

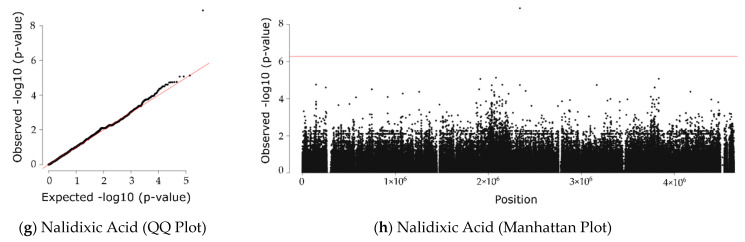
GWAS analysis. Left: QQ plots of observed vs. expected *p*-values show a few highly significant *p*-values. Right: Manhattan plots of chromosomal position vs. *p*-value show mutations passing the Bonferroni-corrected threshold as dots above the red line.

We carried out a GWAS study to determine associations between the high-quality variants and resistance levels of the four quinolones investigated (nalidixic acid, norfloxacin, ciprofloxacin, and levofloxacin). To check for the control of our GWAS over the population structure, we plotted *p*-values expected under randomness against observed *p*-values (see QQ plots in Figure 4). The plots confirm that the correction for population structure was satisfactory, as a deviation from the null hypothesis (the identity line) is only evident at the tail of the plots. Next, we visualized the results of the GWAS using Manhattan plots, which reveal that there are some highly significant variants passing the rigorous Bonferroni-corrected *p*-value (the horizontal line).

In total, we obtained 13 highly significant variants, 3 in *gyrA* (position = 2339162, allele = T, effect = D87N; position = 2339173, allele = A, effect = S83L) and *parC* (position = 3165735, allele = A, effect = S80I) and 10 novel candidate variants in the five genes *bdcA* (position = 4473651, allele = T, effect = G135S), *valS* (position = 4481639, allele = A, effect = R733; position = 4481393, allele = A, effect = N815; position = 4481216, allele = T, effect = E874; position = 4482482, allele = A, effect = D452; position = 4482443, allele = A, effect = V465; position = 4482440, allele = T, effect = L466), *lptG* (position = 4487635, allele = A, effect = V106), *lptF* (position = 4486808, allele = A, effect = Q197), and *ivy* (position = 240711, allele = T, effect = T123) (see Table 2). The variant in *bdcA* leads to an amino acid change, while the remaining nine do not. Across all four quinolones, the mutations in *gyrA* and *parC* ranked highest, thus confirming the validity of the approach taken (Table 2). As shown in the table, the frequency and effect sizes of the novel candidate variants are on a par with the positive controls. This means that the existence of an effect (*p*-value) and the size of the effect (beta) are both given. While all variants pass the Bonferroni-corrected *p*-value threshold (5.21 × 10^−7^), the positive controls exceed it very substantially (Table 2).

### 2.5. Loci of Novel Candidate Variants Are Not in LD with Loci of Positive Controls

**Figure 5:**
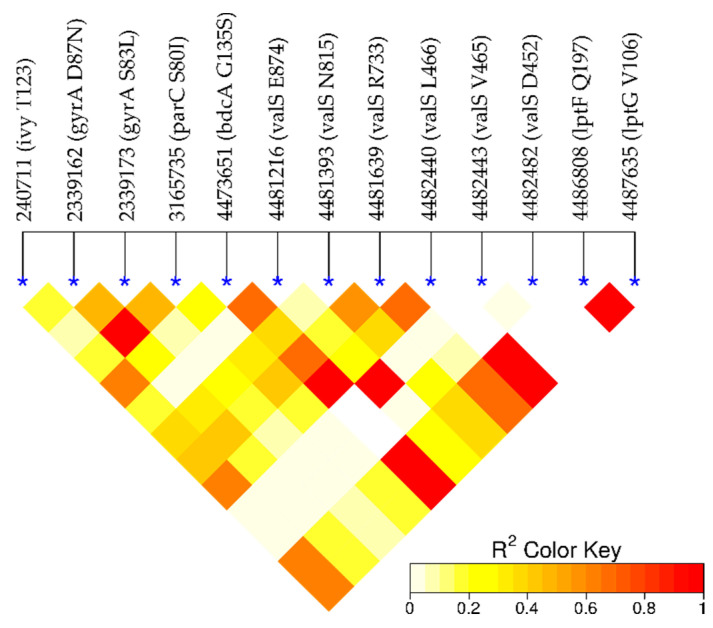
Linkage disequilibrium (LD). High values (red) indicate a dependence of the loci. As expected, the loci in *gyrA* and *parC* are in linkage disequilibrium. Importantly, they are not in LD with the remaining novel candidate loci. Interestingly, there is some dependence within the novel loci, in particular, the locus in *bdcA* is in LD with the loci in *valS*.

To check the independence of the significant variants from one another, we measured the linkage disequilibrium (LD) for the loci of these variants (see Figure 5). The loci of known quinolone resistance-conferring variants, *gyrA* S83L, *gyrA* D87N, and *parC* S80I, are in LD. They are located at the sites where the drugs bind to *gyrA* and *parC* and ensure the correct function of the gene products despite treatment. The loci of 10 novel variants are not in LD with those of known resistance-conferring variants, which suggests that they confer resistance by a different mechanism from *gyrA* and *parC*. Among the novel loci, there are dependencies. In particular, the locus of the nonsynonymous variant in *bdcA* is in LD with loci of synonymous mutations in *valS*. This may mean that these novel variants act in a shared mechanism, which raises the question of whether the biological functions of the novel mutations can be linked to antibiotic resistance.

### 2.6. Biological Function of bdcA

The *bdcA* gene plays a role in biofilm dispersal [20,21], and biofilm formation generally increases antimicrobial resistance [22,23]. It could be hypothesized that a variant in this gene disrupts biofilm dispersal and leads to biofilm formation and resistance. However, while this may happen in nature, it is unclear whether this effect is also present in the disk diffusion assay underlying the present data. This gene is present in nearly all isolates (85–90% in our data and NCBI data), which means that it is close to being a core gene, but that it is not essential for survival.

### 2.7. Biological Function of valS

The *valS* gene product is an aminoacyl-tRNA synthetase (aaRS), which charges tRNA encoding valine with the valine amino acid. The aaRS enzymes are promising targets for antimicrobial development [24,25] as targeting them can inhibit the translation process, cell growth, and finally cell viability. Although aaRS enzymes are not known as direct quinolone targets, there is evidence that nonsynonymous mutations in aaRS enzymes increase ciprofloxacin resistance by upregulating the expression of efflux pumps [26]. In our data, we found synonymous *valS* mutations for ciprofloxacin to just miss satisfying the *p*-value cut-off (Supplementary data). For levofloxacin and norfloxacin, they passed the cut-off. *valS* provides a very basic function and is a core gene present in all isolates.

### 2.8. Biological Function of ivy

The gene product of *ivy* is a strong inhibitor of lysozyme C. Expression of *ivy* protects porous cell-wall *E. coli* mutants from the lytic effect of lysozyme, suggesting that it is a response against the permeabilizing effects of the innate vertebrate immune system. As such, *ivy* acts as a virulence factor for a number of Gram-negative bacteria infecting vertebrates [27].

### 2.9. Biological Function of lptG and lptF

The gene products of *lptG* and *lptF* are part of the ABC transporter complex LptBFG involved in the translocation of lipopolysaccharide from the inner membrane to the outer membrane. Thus, there is no direct connection to antibiotic resistance; however, the link to transport is in line with other resistance mechanisms such as increased expression of efflux pumps [28].

### 2.10. Analyzing the Novel Nonsynonymous Mutation (bdcA G135S)

#### 2.10.1. Structural Analysis

**Figure 6:**
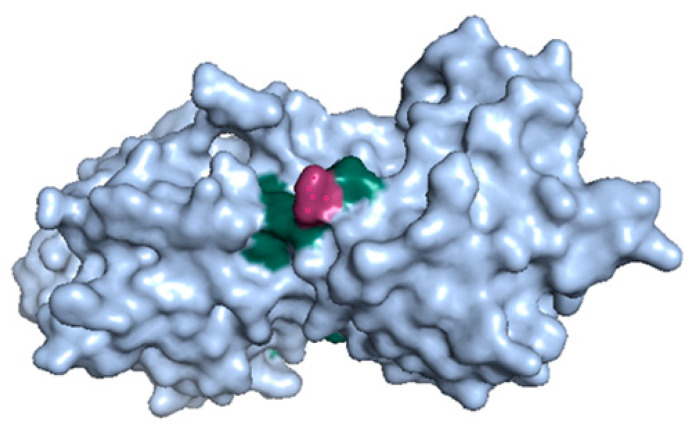
The 3D structure of BdcA (PDB id: 4PCV). The *bdcA* G135S mutation (red) is at the surface and near active site residues Ser132 and Tyr146 (green).

To shed more light on the nonsynonymous variant *bdcA* G135S, we explored its protein structures (Figure 6). The variant Gly135Ser in *bdcA* is in the vicinity of the active site residues Ser132 and Tyr146 [20]. Serine is bigger than glycine, and it may influence a loop formed by the residues 136–144 and thus regulate the active site, which may influence biofilm dispersal.

#### 2.10.2. Variant *bdcA* G135S Wrt. Other Antibiotics

For *bdcA* G135S, we wanted to understand whether its role in antibiotic resistance is limited to quinolones or not. For 16 other antibiotics, there were variants that significantly correlated with resistance (see Supplementary data). For all antibiotics but tobramycin, the *bdcA* mutation is not significant. This suggests that *bdcA* G135S may act independently of fluoroquinolone, which would be consistent with biofilm formation being a general mechanism independent of fluoroquinolone.

#### 2.10.3. Variant *bdcA* Wrt. *E. coli*

Next, we wanted to know whether the prevalence of *bdcA* G135S in our data is representative of other *E. coli* genomes. In 1340 complete *E. coli* genomes available from the NCBI, we could find the *bdcA* gene in 1209 genomes and *bdcA* G135S in 24. Thus, about 2% of genomes carry this mutation, which is slightly less than but still comparable to the 5% present in our data.

#### 2.10.4. Variant *bdcA* Wrt. Other Gammaproteobacteria

BdcA is present in other bacteria. We investigated Gammaproteobacteria, which comprise Pseudomonadaceae besides enterobacteria. We analyzed 152 *bdcA* sequences retrieved from Eggnog 5.0 and found alanine most frequently (65%) and glycine less frequently (24%). Serine appeared in 2% of the species, which may mean that the resistance mechanism is not limited to *E. coli*.

## 3. Discussion

It took around 30 years to move from the discovery of nalidixic acid to the discovery of its targets and mechanism of action. Here, we have shown that sequencing and phenotyping data of a small number of genomes from a single site are sufficient for a GWAS model to reveal the quinolone targets (*gyrA* and *parC*) with a very high statistical significance (*p*-value in the range of 1 × 10^−18^ to 7 × 10^−8^). Besides *gyrA* and *parC*, which passed our *p*-value cut off (5.21 × 10^−7^), we could find mutations in less-studied genes involved in modifying target enzymes, such as *gyrB* and *parE*, as well as in the genes involved in alterations of permeation, such as *acrB*, *ompC*, *mdtK* (*norE*), and *mdfA*, which did not pass our *p*-value cutoff but were mildly significant (*p*-value around 5 × 10^−3^). We believe that having a bigger dataset could lead to a more significant association between mutations in these genes and quinolone resistance.

Furthermore, our GWAS model revealed 10 new mutations, whose significance in relation to quinolone resistance passed our *p*-value cut-off. The most promising mutation is G135S in the biofilm dispersal gene *bdcA*, which is present in nearly all isolates but is not essential for *E. coli* survival [29]. Mapping the *bdcA* mutation onto a protein structure of BdcA revealed its location on the surface of the protein and close to the active site. Hence, this suggests an impact on enzymatic activity, which may influence biofilm dispersion and hence indirectly relate to antibiotic resistance. Ma et al. showed that *E. coli* BdcA controls biofilm dispersal in *Pseudomonas aeruginosa* [30], which were the most abundant Gammaproteobacteria containing *bdcA* in our analysis. This indicates that mutations in *E. coli bdcA* may act indirectly on antibiotic resistance. If BdcA consequently emerges as a novel drug target, then the next steps in drug development could target the active site with residues S132 and Y146, which are in direct proximity to the mutation *bdcA* G135S. Importantly, *bdcA* G135S is a novel candidate resistance mutation as its locus is not in LD with loci of the known mutations in *gyrA* and *parC*.

We found *bdcA* G135S in 5% of the analyzed genomes, which appears in line with a prevalence of 2% in 1209 other *E. coli* genomes obtained from the NCBI. We also checked the presence of these mutations in other Gammaproteobacteria and revealed that *bdcA* is present and well conserved but that the mutation appears specific to *E. coli*. Furthermore, we also checked whether *bdcA* G135S correlates with resistance to non-quinolone antibiotics. This was the case for tobramycin, an aminoglycoside, but not for all other examined antibiotics. Isolates with the *bdcA* G135S mutation belonged to the phylogenetic group A, which is less likely to contain pathogenetic isolates. Phylogroup A is equally abundant in human feces and wastewater [31], which may point to an origin of the mutation in a human rather than a natural environment.

Besides *bdcA* G135S, we found nine synonymous mutations whose mechanism of action is likely to be indirect. Most interesting are the abundant mutations in the aminoacyl-tRNA synthetase *valS*, which has an essential role in protein synthesis and is part of the core genome and therefore present in all isolates. Furthermore, it is classified as an essential gene [29]. It may be a suitable drug target [32] due to its evolutionary divergence between prokaryotic and eukaryotic enzymes and high conservation across different bacterial pathogens, as well as its solubility, stability, and ease of purification. However, since the mutations in *valS* were synonymous, they will not exert a direct structural or functional effect on their gene product but may act indirectly.

In summary, *bdcA* G135S and the discovered silent mutations are statistically significantly correlated with quinolone resistance (*p*-value in the range of 4 × 10^−9^ to 1 × 10^−7^) in wastewater *E. coli*. They appear to be mostly specific to *E. coli* and to quinolones and independent of known resistance-conferring mutations. Further research is needed to corroborate the correlation between these mutations and quinolone resistance and to shed light on the molecular mechanism leading to resistance.

## 4. Materials and Methods

### 4.1. Sampling, Phenotyping, and Sequencing

We collected 1178 *E. coli* isolates from the inflow and outflow of the municipal wastewater treatment plant in Dresden, Germany [19]. The isolates were phenotyped using the agar diffusion method for 20 commonly prescribed antibiotics, including the four quinolones nalidixic acid, norfloxacin, ciprofloxacin, and levofloxacin. Considering the isolates’ resistance to these 20 antibiotics, 103 phenotype-representative isolates were selected for whole-genome sequencing with Illumina MiSeq (available from NCBI’s SRA database, PRJNA380388: https://www.ncbi.nlm.nih.gov/sra/PRJNA380388); see [19] for more details. The unbiased sampling and selection of representative phenotypes were important for the subsequent GWAS analysis, which required both resistant and susceptible isolates.

### 4.2. Sequence Processing and Quality Control

Reads were mapped onto *E. coli* K12 MG1655 with the Burrow-Wheeler Aligner (BWA) v0.7.12 and sorted with Picard v1.105. Variants were called using the genomic analysis toolkit GATK 4.1.1.0 [33] with *E. coli* K12 MG1655 as reference. We filtered variants following standard protocols [34] and the GATK best practices (for SNPs: QD < 2.0, QUAL < 30.0, or FS > 60.0; for INDELs: QD < 2.0, QUAL < 30.0, or FS > 200.0). Variants with low genotype quality (GQ < 20) and variants with high missingness among samples (>15%) were removed. For more details regarding the filtering steps, see Table 1. To analyze the association of each alternative allele separately, variants with multiple alternative alleles were split into multiple records with BCFtools 1.7 [35]. Rare variants with minor allele frequency (MAF) < 5% were excluded using Pyseer 1.3.0. Finally, variants were functionally annotated using SnpEff 4.3T [36].

### 4.3. Pan-variome and Core-variome

In [37,38], a procedure was introduced to compute the pan-genome and core genome. We extended this procedure to calculate the pan-variome and core-variome. The *x*-axis in the pan-variome and core-variome plots (Figure 2) represents the number of randomly selected genomes, from 1 to 99. The *y*-axis shows the size of the union (pan) and intersection (core) of the variants for these randomly selected genomes. It should be noted that for each number of the selected genomes, the process is repeated randomly over 1000 iterations. Afterward, the average and standard deviation for the 1000 iterations are computed.

### 4.4. Phylogenetic Tree and Population Structure

We built a phylogenetic tree from the VCF file with VCF-kit 0.1.6 [39]. To detect the outlier samples, we applied multidimensional scaling (MDS) on the distances in the phylogenetic tree, and four isolates were detected as outliers. These isolates were removed for the subsequent GWAS analysis. Next, the number of important components was determined. To do so, we drew a scree plot for the eigenvalues of the MDS model. The scree plot revealed component number 4 as the knee point. Therefore, we picked four components to be used as covariates for the regression model to control for population structure. To compare the results of the phylogenetic tree, built based on the variant file (VCF file), and the phylogroups, constructed previously [19] based on the classical classification by Clermont et al. [37,40], we visualized the MDS plot using the scatter3d function of the plot3d R package and colored the samples based on the phylogroups. For more verification, we applied hierarchical clustering with a dendrogram on the binary matrix of presence/absence of variants for different samples and a side color based on phylogroups using the heatmap function from the R package stats.

### 4.5. Genome-Wide Association Study (GWAS)

Generalized linear models were developed using Pyseer 1.3.0 [41] to determine the significance of the association between each variant and each antibiotic. To do so, we ran the fixed effects (SEER) model in this package to correlate our antibiotic resistance data (diameter of inhibition zone in disk diffusion method) with the presence/absence of our variants. We also added some covariates to our linear regression model to take the population structure into account (see Section 4.4). To address the problem of multiple comparisons, we calculated a Bonferroni-corrected significance threshold for our GWAS analysis using the same tool. We visualized GWAS results with quantile–quantile (QQ) and Manhattan plots using the R package qqman. We calculated the linkage disequilibrium (LD) between the loci of significant variants using PLINK v1.90b6.10 [42]. The R package LDheatmap [43] was used to visualize LD results.

### 4.6. Analyzing the Novel Nonsynonymous Mutation (bdcA G135S)

The 3D structure of BdcA was retrieved from protein databank PDB (4PCV) and visualized using PyMOL 2.2.0. To check the frequency of *bdcA* G135S in other *E. coli* genomes, we downloaded 1340 *E. coli* genomes from NCBI (https://www.ncbi.nlm.nih.gov/) (accessed on 27 October 2020) and identified the locus in each genome by searching for an exact match of the 10-nucleotide-long sequence ATTCACGGAG, which follows after the locus of the *bdcA* mutation and is conserved across all the retrieved genomes. We also retrieved the multiple sequence alignment ENOG50 1RQ0S for *bdcA* across all Gammaproteobacteria from Eggnog 5.0 [44]. Residue 135 in the ungapped *bdcA* sequence was shifted to position 207 in the gapped multiple sequence alignment.

## Figures and Tables

**Table 1:** Quality control (QC): reduction of some 457,000 raw variants to 206,633 high-quality variants. Filtering the rare variants (based on MAF) is the main filter.

Step	Change	Mutations	Description and Configuration
**1. Variant calling**		457,554	Call germline SNPs and indels via local reassembly of haplotypes Using GATK (HaplotypeCaller) --sample-ploidy 1
**2. Hard filters**	−2%	449,017	Filter the resulting callset Using GATK (VariantFiltration and SelectVariants) For SNPs: --filter-expression ”QUAL < 30.0” Qual is the Phred-scaled probability that a REF/ALT polymorphism exists at this site given sequencing data. --filter-expression ”QD < 2.0” QD is variant confidence (from the QUAL field) normalized by unfiltered depth of variant samples. --filter-expression ”FS > 60.0” FS is the strand bias estimated using Fisher’s exact test. For INDELs: --filter-expression ”QUAL < 30.0” --filter-expression ”QD < 2.0” --filter-expression ”FS > 200.0”
**3. Filtering by GQ and** **missingness**	−15%	382,922	Filter variants with low-quality assigned genotype (GQ) and high missingness (>15%) Using GATK (VariantFiltration and SelectVariants) --filter-expression ”GQ < 20” --max-nocall-fraction 0.15
**4. Splitting alternative alleles**	+8%	413,283	Split variants with multiple alternative alleles into multiple records Using BCFtools norm –m
**5. Filtering by MAF**	−50%	206,633	Exclude rare variants with minor allele frequency (MAF < 5%) Using Pyseer --min-af 0.05

**Table 2:** Mutations significantly correlating with quinolone resistance. The dotted line separates synonymous and nonsynonymous variants. Freq. is the relative frequency among isolates, beta is the effect size, and SE is the standard error of the fit on beta. Effect size is similar for all, and *p*-values differ.

Quinolone	Position	Allele	Gene	Effect	Freq.	Beta	SE	Call Rate	*p*-Value
	3165735	A	*parC*	S80I	0.08	−1.56	0.20	100%	2.43 × 10^−12^
	2339162	T	*gyrA*	D87N	0.08	−1.56	0.20	100%	2.43 × 10^−12^
	2339173	A	*gyrA*	S83L	0.15	−1.20	0.16	99%	4.47 × 10^−12^
	4473651	T	*bdcA*	G135S	0.05	−1.58	0.29	90%	1.35 × 10^−7^
**Levofloxacin**	4481639	A	*valS*	R733	0.07	−1.15	0.24	100%	4.09 × 10^−9^
	4481393	A	*valS*	N815	0.12	−1.11	0.20	100%	6.79 × 10^−8^
	4481216	T	*valS*	E874	0.16	−1.61	0.29	100%	7.09 × 10^−8^
	4482482	A	*valS*	D452	0.05	−1.58	0.29	100%	1.35 × 10^−7^
	4482443	A	*valS*	V465	0.05	−1.58	0.29	100%	1.35 × 10^−7^
	4482440	T	*valS*	L466	0.05	−1.58	0.29	100%	1.35 × 10^−7^
	4486808	A	*lptF*	Q197	0.05	−1.58	0.29	100%	1.35 × 10^−7^
	4487635	A	*lptG*	V106	0.05	−1.58	0.29	100%	1.35 × 10^−7^
	3165735	A	*parC*	S80I	0.08	−2.29	0.22	100%	1.10 × 10^−18^
	2339162	T	*gyrA*	D87N	0.08	−2.29	0.22	100%	1.10 × 10^−18^
	2339173	A	*gyrA*	S83L	0.15	−1.59	0.19	99%	9.25 × 10^−14^
	4473651	T	*bdcA*	G135S	0.05	−2.01	0.36	90%	7.56 × 10^−8^
**Norfloxacin**	4481639	A	*valS*	R733	0.07	−1.85	0.30	100%	5.24 × 10^−9^
	4481216	T	*valS*	E874	0.16	−2.03	0.35	100%	4.36 × 10^−8^
	4481393	A	*valS*	N815	0.12	−1.39	0.25	100%	5.40 × 10^−8^
	4482482	A	*valS*	D452	0.05	−2.01	0.36	100%	7.56 × 10^−8^
	4482443	A	*valS*	V465	0.05	−2.01	0.36	100%	7.56 × 10^−8^
	4482440	T	*valS*	L466	0.05	−2.01	0.36	100%	7.56 × 10^−8^
	4486808	A	*lptF*	Q197	0.05	−2.01	0.36	100%	7.56 × 10^−8^
	4487635	A	*lptG*	V106	0.05	−2.01	0.36	100%	7.56 × 10^−8^
	240711	T	*ivy*	T123	0.05	−2.00	0.36	100%	1.04 × 10^−7^
	3165735	A	*parC*	S80I	0.08	−1.90	0.25	100%	7.37 × 10^−12^
**Ciprofloxacin**	2339162	T	*gyrA*	D87N	0.08	−1.90	0.25	100%	7.37 × 10^−12^
	2339173	A	*gyrA*	S83L	0.15	−1.22	0.22	99%	7.13 × 10^−8^
**Nalidixic acid**	2339173	A	*gyrA*	S83L	0.15	−1.57	0.24	99%	1.32 × 10^−9^

## Data Availability

The data for 103 sequenced samples is available from NCBI’s SRA database, PRJNA380388: https://www.ncbi.nlm.nih.gov/sra/PRJNA380388.

## Supplementary Materials

The following are available online at https://www.mdpi.com/article/10.3390/ijms22116063/s1, Supplementary data, spreadsheets, includes the top 100 most significantly associated variants for 20 commonly prescribed antibiotics. The results for each antibiotic are shown in one separate sheet.
